# Perceptions of the activity, the social climate, and the self during group exercise classes regulate intrinsic satisfaction

**DOI:** 10.3389/fpsyg.2015.01236

**Published:** 2015-08-19

**Authors:** Jaclyn P. Maher, Jinger S. Gottschall, David E. Conroy

**Affiliations:** ^1^Department of Kinesiology, Pennsylvania State University, University ParkPA, USA; ^2^Department of Preventive Medicine, University of Southern California, Los AngelesCA, USA; ^3^Department of Preventive Medicine, Northwestern University, ChicagoIL, USA

**Keywords:** cohesion, instructor style, competence, intensity, structured-exercise

## Abstract

Engaging in regular physical activity is a challenging task for many adults. Intrinsic satisfaction with exercise classes is thought to promote adherence to physical activity. This study examined the characteristics of exercise classes that impact within-person changes in intrinsic satisfaction over the course of an extended group exercise program. A 30-week physical activity trial was conducted with assessments at the end of each class. Community-living adults (*n* = 29) were instructed to complete at least six group exercise classes each week and, following each exercise class, complete a questionnaire asking about the characteristics of the class and the participant’s evaluation of the class. Intrinsic satisfaction was high, on average, but varied as much within-person from class-to-class as it did between exercisers. Participants reported the greatest intrinsic satisfaction when classes placed greater emphasis on exercisers’ involvement with the group task, feelings of competence, and encouragement from the instructor. For the most part, exercise classes that were more intense than usual were perceived by exercisers as less intrinsically satisfying. Some overall characteristics of the exercise classes were also associated with intrinsic satisfaction. The social and motivational characteristics of group exercise classes contribute to exercisers’ intrinsic satisfaction with classes and attention to those dynamics, as well as the intensity of the exercise, may improve adherence for exercise regimens.

## Introduction

Engaging in regular physical activity is associated with an assortment of positive physical and psychological health benefits (e.g., reduced risk of cardiovascular disease, type II diabetes, some types of cancer, and improved mental health; Physical Activity Guidelines Advisory Committee, 2008). Despite these well-known benefits, many people remain inactive. In America, for example, recent data from the National Health Interview Survey indicate that less than half of Americans are meeting current physical activity guidelines (Carlson et al., 2010). Additionally, a staggering 30% of Americans engage in no leisure-time physical activity, leaving a large percentage of the American population susceptible to chronic conditions associated with inactivity (e.g., obesity, cardiovascular disease, diabetes). These trends regarding inactivity are present in other developed countries as well (e.g., Hallal et al., 2012).

It is often difficult for individuals starting a new program to adhere to exercise regimens and as many as 50% of people who start a new exercise program are thought to drop out within the first 6 months (Armitage, 2005). One reason for the limited success in the initiation of and adherence to behavior change is that health promotion programs often rely of social cognitive theories of motivation, which have limited predictive power concerning physical activity, to inform program development (McEachan et al., 2011; Rhodes and Dickau, 2012). These theories focus exclusively on the individual’s thoughts or cognitions regarding exercise and fail to consider influences extending beyond the individual (such as the exercise environment) or what factors contribute to exercise being intrinsically satisfying. Satisfaction has been proposed as a determinant of health behavior adherence and changes in satisfaction have predicted adherence to an exercise-based rehabilitation program (Fleig et al., 2011). Group exercise classes are one popular form of exercise that many individuals use to increase their activity yet little is known about factors that influence satisfaction within those classes. In this study, we examined the factors that influenced exercisers’ evaluation of class satisfaction in a 30-week fitness trial for adults who do not engage in regular physical activity.

Satisfaction implies progress toward an individual’s goals (Sheldon and Elliot, 1999). Goals relevant to participating in an exercise class can be characterized as intrinsic or extrinsic (Kasser and Ryan, 1996). Intrinsic goals, for example, focus on the pursuit of enjoyment, fun, or satisfaction whereas extrinsic goals, for example, involve the pursuit of weight loss, or improved lipid profiles. For the purposes of this study we focused exclusively on the intrinsic satisfaction derived from exercise classes. Intrinsic satisfaction with an exercise class refers to the class being appraised favorably, as reflected by participants’ perceptions that an activity is interesting, enjoyable, and challenging, and competence motivation can influence these perceptions directly (Elliot and Dweck, 2013).

There has been a large body of literature documenting the processes that influence intrinsic satisfaction; however, this body of work has largely relied on cross-sectional comparisons of people who are more or less satisfied exercisers and the factors that are associated with between-person differences in satisfaction (e.g., Fox et al., 2000; Edmunds et al., 2008). The studies that have gone beyond cross-sectional comparisons have focused on time-structured changes documenting pre-to-post changes over a season, exercise program, semester, year, etc. (e.g., Cresswell and Eklund, 2005). Given that domain-specific evaluations of satisfaction change within people over time (Heller et al., 2006), intrinsic satisfaction with exercise classes may also be dynamic, fluctuating from class-to-class depending on the quality of people’s experiences in each class. People’s day-to-day experiences within exercise classes should impact their evaluations and subsequent motivation toward the classes. We propose that dynamic perceptions of the activity, the social climate, and the self during exercise classes regulate fluctuations in exercisers’ intrinsic satisfaction with those classes.

Typically, influences on intrinsic satisfaction are examined in terms of time-invariant, between-person processes that represent stable characteristics of exercise classes. The dynamic contexts of individual exercise classes (e.g., having a class that is too challenging or an instructor who is too highly encouraging) represent time-varying, within-person influences on exercisers’ intrinsic satisfaction. By simultaneously examining time-invariant and time-varying processes this study can shed light on global ways to enhance intrinsic satisfaction (via time-invariant processes) as well as ways to enhance satisfaction from class-to-class or day-to-day (via time-varying processes). This may lead to greater adherence to exercise programs on a class-to-class or day-to-day basis, which could, in turn, have implications for adherence over the long-term. To that end, this study was designed to investigate three general influences on intrinsic satisfaction with group exercise classes: perceptions of the activity, the social climate, and the self.

Regarding perceptions of the activity, the intensity of an exercise class may influence an exerciser’s intrinsic satisfaction with the class. Exercising at high intensities often results in unpleasant feelings during exercise for novice or untrained exercisers (Ekkekakis, 2003). To the extent that these affective responses are inconsistent with hedonic goals, more intense exercise classes (i.e., greater perceived exertion, maximum heart rate) should be associated with lower intrinsic satisfaction. Furthermore, the intensity of exercise classes is likely to vary from class-to-class as a function of the activities performed and the physical demand placed on exercisers. The association between intensity and intrinsic satisfaction may be confounded by the type of activity during the class. For example, cardio, strength, and flexibility classes vary in their physical demands and required abilities. These activity differences may influence intrinsic satisfaction by moderating how intensity covaries with intrinsic satisfaction. Individuals attending cardio or strength classes are more likely to experience discomfort (either by approaching the ventilatory threshold or experiencing muscle strain or fatigue) due to the physical demands of these strenuous classes (Acevedo et al., 1994; Ekkekakis, 2003). Therefore the more strenuous these classes are, the less likely individuals are to find intrinsic satisfaction within these classes. Whereas individuals attending flexibility classes, which generally focus on a structured series of stretches and poses, are likely to be less challenging physically. Therefore when flexibility classes require greater amounts of exertion, participants are able to meet these demands without excessive discomfort or strain, resulting in greater satisfaction. For this study, we hypothesized that perceived exercise intensity and intrinsic satisfaction would be negatively associated in cardio and strength training classes but positively associated in flexibility classes; these hypotheses were identical for within-person (time-varying) and between-person (time-invariant) associations.

Regarding the climate, social processes at the group and individual levels serve as key determinants of participation and performance in an exercise setting (Buckworth and Dishman, 2007). Feelings of social connection are strongly and positively associated with feelings of intrinsic satisfaction (Sheldon et al., 2001). Within the context of exercise classes, both perceived cohesion and instructor style are likely to contribute to the social climate.

Cohesion is “a dynamic process that is reflected in the tendency for a group to stick together and remain united in pursuit of its instrumental objectives and/or for the satisfaction of member affective needs” (Carron et al., 1998, p. 213). In cross-sectional studies, perceived cohesion has been linked with greater enjoyment, intrinsic satisfaction, and ultimately adherence (a time-invariant process; Spink and Carron, 1994; Carron et al., 1998; Estabrooks and Carron, 1999; Fraser and Spink, 2002). Few studies have examined how cohesion changes over time (Dunlop et al., 2012) and we are not aware of any examining how changes in cohesion influence changes in intrinsic satisfaction. For this study, we simultaneously investigated how changes in perceived class cohesion were associated with exercisers’ intrinsic satisfaction (a time-varying process) as well as how average levels of perceived cohesion were associated with exercisers’ intrinsic satisfaction across classes (a time-invariant process). We hypothesized that perceived cohesion during a class would be positively associated with intrinsic satisfaction with the class at both the between- and within-person levels.

An exercise instructor’s teaching style is another aspect of the social climate that can influence exercisers’ enjoyment of and intrinsic satisfaction with an exercise class. In two randomized control trials, exercisers reported greater enjoyment in classes with instructors who used more encouragement, positive reinforcement, and individualized feedback than in classes with instructors who had a bland teaching style (Fox et al., 2000). Novice exercisers have also rated their enjoyment of exercise classes higher when instructors were more encouraging as opposed to when instructors were devoid of any enthusiasm and only provided feedback on the technical nature of the exercises (Bray et al., 2005). The fact that exercisers found technical feedback less enjoyable could indicate that exercisers prefer a less controlling social climate in classes. Given the potential for instructor’s behavior to change from class-to-class, intensive longitudinal analysis is needed to determine the association between instructor style and fluctuations in intrinsic satisfaction with exercise classes. In this study, we hypothesized that instructor encouragement would be positively associated with intrinsic satisfaction at both the between- and within-person level, and instructor control would be negatively associated with intrinsic satisfaction at both the between- and within-person level.

Regarding perceptions of the self, perceived competence is one motivational belief that likely impacts evaluations of an exercise class. Perceived competence refers to an individual’s belief that they are capable of being effective at a given activity (Elliot and Dweck, 2013). Consistent with competence theories of motivation, perceptions of competence satisfy a basic need and facilitate goal attainment by increasing persistence toward goals (Elliot and Dweck, 2013). For example, Whitehead and Corbin (1991) found that increases in high school students’ perceived competence after running led to increased enjoyment, interest, and effort. Perceived competence is likely to vary across contexts and across time as activities change. It is presently unclear how changes in perceived competence influence intrinsic satisfaction with exercise classes. For this study, we hypothesized that both the time-invariant and time-varying associations between perceived competence and intrinsic satisfaction would be positive.

A 30-week physical activity trial was conducted with adults who did not engage in regular physical activity prior to enrolling. This manuscript serves as a secondary analysis of that data designed to better understand the factors that influence fluctuations in intrinsic satisfaction across exercise classes. Whereas previous research has focused on comparisons between more- and less-satisfied exercisers (i.e., time-invariant associations), this study is intended to capture the dynamics of intrinsic satisfaction and evaluate the time-invariant and time-varying processes that influence intrinsic satisfaction over the course of a group exercise program. Three sets of hypotheses were made regarding perceptions of the activity, the social climate, and the self. First, we hypothesized that the time-invariant and time-varying associations between exercise intensity and intrinsic satisfaction would be negative in cardio and strength training classes but that these associations would be positive in flexibility classes. Second, we hypothesized that exercisers would be more intrinsically satisfied when they perceived (a) classes as being more cohesive overall and more cohesive than is typical for a given class, and (b) instructors as being more encouraging and less controlling overall and more encouraging and less controlling than is typical for a given instructor. Finally, we hypothesized that exercisers would be more intrinsically satisfied in classes when they felt more competent with class activities on average and more competent with class activities than is typical for a given class.

## Materials and Methods

### Participants

Thirty-two individuals were recruited from the community to participate in a 30-week physical activity trial. This sample consisted of 18 women and 11 men with a mean age of 30.8 years (SD = 3.7). Participants were mostly Caucasian (75% Caucasian, 19% African American, 6% identified as other) and not Latino/Hispanic (94%). Participants in the sample had body mass index values that ranged from 20.9 to 39.8 kg/m^2^ (*M* = 29.4, SD = 5.4). The majority of the sample was classified as overweight (31%) or obese (45%). Inclusion criteria for participating in the exercise intervention were as follows: participants had to be (a) able to read and understand English, (b) between 25 and 40 years of age, (c) insufficiently active (i.e., engaging in less than 30 min of physical activity per week) for 6 months prior to entering the study, (d) healthy, to the extent that they were free from any heart conditions (e.g., coronary heart disease, cardiac rhythm disturbance, heart transplant) or any other conditions that might be exacerbated by exercising (e.g., asthma, joint problems), and (e) not taking any medications that would limit their physical activity (e.g., beta-blockers, nitrates).

### Procedures

Community-dwelling participants were recruited for this study via flyers placed around the community. Interested participants were first screened via a telephone interview to determine age, current exercise, and availability. Next participants completed a physical screening at the Clinical Research Center on campus which involved a screening exam with height, weight, blood pressure, blood draw, and an IDXA in addition to a medical history report. Eligible participants were then scheduled to attend an initial lab session at a research lab on the university’s campus during which a research assistant described the study and participants provided informed consent. Following this initial lab session, participants began attending classes at a community fitness facility as part of 6-weeks familiarization period.

The fitness facility offered six different hour-long exercise classes that participants were allowed to attend. Classes offered included four cardio classes, one strength training class, and one flexibility class. As participants became acclimated to the exercise classes, participants were asked to steadily increase the number of classes they attended so that by the beginning of week 7 participants would be able to complete six exercise classes per week (i.e., three cardio, two strength, one flexibility). At the beginning of week 7 participants were instructed to continue attending six classes per week until week 18, at which point participants were instructed to start attending one more cardio class each week. Participants were also given a Polar RS400 heart rate monitor to wear during each class beginning at week 7.

Following each exercise class participants completed a questionnaire asking about the characteristics of the class and the participant’s feelings toward the class. Due to the intensive sampling protocol, we were concerned about burdening or fatiguing participants. To reduce the potential impact of these threats, we altered original measures or created single-item measures focused on each construct of interest. Participants were prompted to reflect on the class they had *just completed*. Study procedures were approved by the local Institutional Review Board.

### Measures

#### Intrinsic Satisfaction with Exercise Class

*Intrinsic satisfaction with exercise class* was assessed using three items. These three items included: interest, enjoyment, and challenge. Interest was assessed using a single item. Participants were given the stem, “This class was,” and responded on a 1 (*uninteresting*) to 7 (*interesting*) scale. Enjoyment was assessed using the single-item, “I enjoyed this class,” and participants responded using a 1 (*not true of me at all*) to 7 (*extremely true of me*) scale. Challenge was assessed using the single-item, “I viewed this class as a positive challenge,” and participants responded using a 1 (*not true of me at all*) to 7 (*extremely true of me*) scale. Responses to the intrinsic satisfaction with exercise class items were strongly correlated (*M*_correlation_ = 0.72, range = 0.60–0.86) and the internal consistency of the three items was 0.92 (across all occasions and persons). A composite *intrinsic satisfaction with exercise class* score was created by averaging the scores of the three aforementioned items.

#### Exercise Intensity

Two measures of exercise class intensity were collected. *Perceived exertion* was measured using the Rating of Perceived Exertion Scale (Borg, 1998). With instructions to, “Rate how hard you had to exert yourself during the exercise class you just completed. Focus on your total feeling of exertion. Do not focus on just one factor such as shortness of breath or leg pain,” participants rated their perceived exertion on a 6 (*no exertion at all*) to 20 (*maximum exertion*) scale.

*Maximum heart rate* was determined using Polar RS400 heart rate monitors (Lake Success, NY, USA). Within the range of heart rate measurements taken during the exercise class, maximum heart rate was calculated based on the top ten percent of heart rate measurements. This upper range of heart rate measurements was then averaged to create a single score for each exercise class.

#### Cohesion

Cohesion was assessed using three items from the Physical Activity Group Environment Questionnaire (Estabrooks and Carron, 2000). These items were drawn from scales for *attraction to group task, attraction to group social, and group interaction social* (we omitted group integration task *a priori* because the available items were not well-matched to the structure of this supervised exercise program). Participants rated each cohesion item [e.g., “I liked the amount of physical activity I got in this class” (attraction to group task)] on a 1 (*strongly disagree*) to 7 (*strongly agree*) scale. We chose to include these three elements of cohesion as individual predictors of intrinsic satisfaction given that attraction to group task, attraction to group social, and group interaction social represent distinct aspect of cohesion (Estabrooks and Carron, 2000).

#### Instructor Behavior

Characteristics of instructor’s behavior were assessed using two single-item measures modified from Conroy and Coatsworth (2007). *Encouragement* and *control* were assessed with the items, “The instructor encouraged me,” and “The instructor made me do things their way,” respectively. Participants rated these items using a 1 (*strongly disagree*) to 7 (*strongly agree*) scale.

#### Perceived Competence

Perceived competence was measured using three items, which were created for the purpose of this study. Each item represented a different aspect of competence (i.e., task-referenced competence [“I believe I completed the exercises today the way they should be done”], self-referenced competence [“I believe I improved today compared to my past performances in the class”], and normatively referenced competence [“I believe I did well today compared to my peers in the class”]). Participants rated each of these three items on a 1 (*not true of me at all*) to 7 (*extremely true of me*) scale. Items were highly correlated (*r*s ≥ 0.71) and the internal consistency of items was 0.89 (across all occasions and persons). Responses to the three perceived competence items were averaged to create a perceived competence score. Amiot et al. (2004) previously used a similar approach to assess perceived competence.

### Data Analysis

Classes were nested within people in this study so we used multilevel modeling to test our hypotheses about between- and within-person associations with characteristics of the class and intrinsic satisfaction with the exercise class (Snijders and Bosker, 1999). Models were estimated using SAS 9.3 PROC MIXED (Littell et al., 1996), with the small amount of missing observations (<7%; *n_observations_* = 247) treated as missing completely at random. A multilevel model was estimated to determine the unique influence of social and motivational processes as well as physical demand on exercise class intrinsic satisfaction while controlling for time-varying factors such as time in study and day of week and the time-invariant factors, BMI. A Pseudo-R^2^ was also estimated for each predictor of intrinsic satisfaction to provide an indicator of effect size for each potential influence (Snijders and Bosker, 1999).

#### Data Preparation

Our primary interest was in the within-person associations between class characteristics (i.e., intensity, social, and motivational processes) and intrinsic satisfaction so we person-centered scores for each predictor to separate between- and within-person components (i.e., time-invariant and time-varying components, respectively; Schwartz and Stone, 1998; Bolger et al., 2003). For example, person *i*’s overall feelings of perceived competence (*Overall Perceived Competence_i_*) was calculated as the within-person mean of her responses across all of the classes she attended and each person’s class-specific perceived competence (*Class Perceived Competence_ci_*) was calculated as the deviation of class *c*’s score from the individual mean. Therefore, all class-level scores (e.g., class perceived competence) are class-specific deviations from the individual’s average across all classes they rated.

In addition to separating between- and within-person components, dummy variables were created to represent different types of classes. Cardio classes were most frequent so they served as the reference category. Intrinsic satisfaction with exercise class may change as participants become more familiar with classes and regimens so we also created a linear time variable to control for changes over the course of the study. Finally, the social calendar (e.g., work schedules, leisure time availability) may play a role in participants’ intrinsic satisfaction with classes so we created six dummy variables to control for these systematic differences in intrinsic satisfaction. Tuesday was selected as the reference category because classes held on that day were rated as most satisfying.

## Results

Of the thirty-two individuals that met eligibility criteria and enrolled in the study, three discontinued participation prior to the start of the study. Of the 29 participants that began the study, all participants remained in the study up to the half way point, but only 25 participants completed the entire study. Reasons for drop out included medical issues (*n* = 2), schedule conflict (*n* = 1), and moving out of area (*n* = 1). Participants attended and provided data for a total of 3,698 of the 4,524 possible person-classes (82% response rate) and 55% of participants (*n* = 16) reported on at least 139 of the 156 possible classes (Median = 140 classes, *M* = 127.52, SD = 32.49). Descriptive statistics are displayed in **Table 1**. On average, participants reported high levels of intrinsic satisfaction with exercise classes (*M* = 6.36 out of 7, SD = 0.69). Participants also tended to experience moderate amounts of cohesion within classes (*M* ranged from 4.93 to 6.56 out of 7, SD ranged from 0.90 to 1.61). Instructors were perceived as highly encouraging (*M* = 6.71 out of 7, SD = 0.72) and moderately controlling (*M* = 4.23 out of 7, SD = 2.35). On average, participants also felt moderately competent during exercise classes (*M* = 5.67 out of 7, SD = 1.13). Finally, participants appeared to find the exercise classes to be physically demanding (*M*_Percieved Exertion_ = 16.61 out of 20, SD = 2.32; *M*_Maximum Heart Rate_ = 154.28, SD = 9.09).

**Table 1:** Descriptive statistics, correlations, and intraclass correlations of intrinsic satisfaction with exercise class and predictor variables.

	*M*	SD	1	2	3	4	5	6	7	8	9
(1) Satisfaction with Exercise Class	6.36	0.69	(0.56)	0.88	0.63	0.04	0.76	0.11	0.54	0.32	-0.24
(2) Attraction to Group – Task	6.56	0.90	0.75	(0.51)	0.59	0.06	0.78	-0.09	0.40	0.22	-0.20
(3) Attraction to Group – Social	5.42	1.35	0.42	0.39	(0.43)	0.67	0.65	-0.01	0.69	-0.12	-0.13
(4) Group Interaction – Social	4.93	1.61	0.08	0.07	0.55	(0.57)	0.22	0.16	0.43	-0.32	-0.26
(5) Instructor Encouragement	6.71	0.72	0.65	0.52	0.27	0.11	(0.50)	-0.04	0.44	0.19	-0.40
(6) Instructor Control	4.23	2.35	0.05	-0.05	0.02	0.18	-0.03	(0.89)	-0.01	0.17	-0.07
(7) Competence	5.67	1.13	0.47	0.39	0.45	0.35	0.28	-0.02	(0.49)	-0.11	0.09
(8) Perceived Exertion	16.61	2.32	0.21	0.24	-0.02	-0.12	0.17	0.14	-0.01	(0.52)	-0.13
(9) Maximum Heart Rate	154.28	9.09	0.03	0.10	0.06	0.01	-0.06	-0.04	0.20	0.25	(0.19)

*Intraclass correlation coefficients representing the proportion of between-person variance appear in parentheses on the diagonal of the correlation matrix. Coefficients below the diagonal are correlations across classes and people. Coefficients above the diagonal represent correlations of within-person means. *M* = sample-level mean, SD = sample-level standard deviation.*

**Table 1** presents two types of bivariate correlations. The first type (below the diagonal) represents correlations across all people and all classes (i.e., ignoring the nesting of classes within people). The second type (above the diagonal) represents correlations among within-person means (i.e., an individual’s average across 156 classes; ignoring variability in ratings). Due to the limitations of both types of correlations, we interpret these correlations descriptively rather than inferentially. Perceived exertion had a moderate, positive correlation with intrinsic satisfaction with exercise class. Maximum heart rate was weakly, and sometimes negatively correlated with intrinsic satisfaction (*r*s = 0.03; -0.24). Intrinsic satisfaction had a moderate, positive correlation with both attraction to group task and social (*r*s ≥ 0.42) whereas intrinsic satisfaction with exercise class has a weak, positive correlation with group integration – social (*r*s = 0.08; 0.04). Instructor encouragement had a moderate to strong positive correlation with intrinsic satisfaction (*r*s = 0.65; 0.76) whereas instructor control had a weak, positive correlation (*r*s = 0.05; 0.11). Perceived competence had moderate, positive correlations with intrinsic satisfaction (*r*s = 0.47; 0.54). Intraclass correlations, which indicate the relative proportion of between- and within-person variability, revealed that measures differed in their proportion of between-person variation over time (*ICC*s ranged from 0.19 to 0.89). Approximately half of the variability in intrinsic satisfaction scores was between-person, which pointed to the need to distinguish between factors that influence between-person and within-person differences in intrinsic satisfaction.

### Multilevel Model of Intrinsic Satisfaction with Exercise Classes

A multilevel model was used to determine the factors that influence intrinsic satisfaction with exercise classes. Results from the multilevel model are displayed in **Table 2**. Concerning time-varying (within-person) influences on intrinsic satisfaction, class-to-class changes in perceived exertion during cardio classes were positively associated with the exercise class being perceived as more intrinsically satisfying, so that classes which exercisers perceived as more exerting than usual were also perceived as more satisfying (γ_10_ = 0.02, *p* < 0.001). Whereas class-to-class changes in perceived exertion during flexibility classes were associated with the class being perceived as less intrinsically satisfying (γ_30_ = -0.02, *p* < 0.001) and class-to-class changes in perceived exertion during cardio and strength classes were unrelated to intrinsic satisfaction (γ_20_ = -0.01, *p* = 0.99). During cardio classes, when participants’ maximum heart rate was higher than usual, they evaluated the class as being more intrinsically satisfying (γ_40_ = 0.01, *p* < 0.001), but during strength classes (γ_50_ = -0.01, *p* < 0.05), when exercisers’ maximum heart rate were higher than usual, they viewed the class as less intrinsically satisfying. Class-to-class changes in maximal heart rate during flexibility classes were not associated with intrinsic satisfaction with the exercise class (γ_60_ = -0.01, *p* = 0.74).

**Table 2:** Results from multilevel model examining between-person and within-person associations of predictor variables with intrinsic satisfaction.

	Parameter estimate (SE)
**Fixed effects**	
Intercept, γ_00_	3.05^∗^ (0.34)
Overall Perceived Exertion, γ_01_	0.01 (0.02)
Class Perceived Exertion, γ_10_	0.02^∗^ (0.01)
Class Perceived Exertion × Strength Class, γ_20_	-0.01 (0.01)
Class Perceived Exertion × Flexibility Class, γ_30_	-0.02^∗^ (0.01)
Overall Maximum Heart Rate, γ_02_	-0.01^∗^ (0.01)
Class Maximum Heart Rate, γ_40_	0.01^∗^ (0.01)
Class Maximum Heart Rate × Strength Class, γ_50_	-0.01^∗^ (0.01)
Class Maximum Heart Rate × Flexibility Class, γ_60_	-0.01 (0.01)
Overall Attraction to Group – Task, γ_03_	0.37^∗^ (0.06)
Class Attraction to Group – Task, γ_70_	0.19^∗^ (0.03)
Overall Attraction to Group – Social, γ_04_	0.22^∗^ (0.06)
Class Attraction to Group – Social, γ_80_	-0.01 (0.01)
Overall Group Interaction – Social, γ_05_	-0.20^∗^ (0.03)
Class Group Interaction – Social, γ_90_	-0.01 (0.01)
Overall Instructor Encouragement, γ_06_	0.08 (0.08)
Class Instructor Encouragement, γ_100_	0.17^∗^ (0.03)
Overall Instructor Control, γ_07_	0.03^∗^ (0.01)
Class Instructor Control, γ_110_	-0.01 (0.03)
Overall Competence, γ_08_	0.07 (0.06)
Class Competence, γ_120_	0.10^∗^ (0.02)
Time in Study, γ_130_	0.01 (0.01)
Monday, γ_140_	-0.02 (0.01)
Wednesday, γ_150_	-0.03^∗^ (0.01)
Thursday, γ_160_	-0.01 (0.01)
Friday, γ_170_	-0.01 (0.01)
Saturday, γ_180_	-0.01 (0.01)
Sunday, γ_190_	-0.05^∗^ (0.02)
Strength Class, γ_200_	0.51^∗^ (0.18)
Flexibility Class, γ_210_	0.56^∗^ (0.19)
BMI, γ_09_	-0.01 (0.01)
**Random effects**	
Variance Intercept, σ^2^*_u_*_0_	2.58^∗^ (0.79)
Variance Attraction to Group – Task, σ^2^*_u7_*	0.02^∗^ (0.01)
Variance Instructor Encouragement, σ^2^*_u_*_10_	0.02^∗^ (0.01)
Variance Competence, σ^2^*_u_*_12_	0.02^∗^ (0.01)
Residual, σ^2^*_e_*	0.06^∗^ (0.01)
-*2LL*	296.3
*AIC*	318.3

*Unstandardized estimates and standard errors. Model is based on 153 occasions nested within 29 participants for a total of 3,451 observations. -*2LL* = -2 Log Likelihood. *AIC* = Akaike Information Criterion. ^∗^*p* < 0.05.*

Exercise classes that emphasized participants’ personal involvement with the group task more than usual, also tended to be perceived by exercisers as more intrinsically satisfying (attraction to group – task; γ_70_ = 0.19, *p* < 0.001). Exercise classes which emphasized participants’ personal involvement in the group socially (attraction to group – social; γ_80_ = -0.01, *p* = 0.99) or emphasized closeness or bonding among class members more than usual (group interaction – social; γ_90_ = 0.01, *p* = 0.28) were not associated with intrinsic satisfaction. Results revealed a positive, within-person association between instructor encouragement and intrinsic satisfaction (γ_100_ = 0.17, *p* < 0.001): exercisers perceived classes as more intrinsically satisfying on days when they perceived their instructors as more encouraging than usual. Class-to-class fluctuations in instructor control were unrelated to intrinsic satisfaction with exercise classes (γ_110_ = -0.01, *p* = 0.32). Exercise classes in which participants felt more competent than usual, were perceived as more intrinsically satisfying (γ_120_ = 0.10, *p* < 0.001).

Concerning time-invariant (between-person) influences on intrinsic satisfaction, results revealed a negative, between-person association between intrinsic satisfaction with exercise class and maximum heart rate (γ_02_ = -0.01, *p* < 0.05). On average, exercisers who achieved higher maximal heart rates during their exercise classes perceived their classes as less intrinsically satisfying. Overall perceived exertion was unrelated to intrinsic satisfaction with exercise classes (γ_01_ = 0.01, *p* = 0.43).

All three domain specific elements of cohesion tested in this study were significantly associated with intrinsic satisfaction at the between-person level. On average, exercise classes that emphasized participants’ personal involvement with the group task (attraction to group – task; γ_03_ = 0.37, *p* < 0.001) or emphasized participants’ personal involvement in the group socially (attraction to group – social; γ_04_ = 0.22, *p* < 0.05) more, on average, also tended to be perceived by exercisers as more intrinsically satisfying. Conversely, exercise classes that tended to emphasize similarity, closeness, and bonding between exercisers more, on average, tended to be perceived as less intrinsically satisfying overall (group integration – social; γ_05_ = -0.15, *p* < 0.001). Exercise classes during which instructors exercised greater control were viewed as more intrinsically satisfying than classes during which, instructors were not controlling (γ_07_ = 0.03, *p* < 0.05). In contrast, overall instructor encouragement was unrelated to intrinsic satisfaction (γ_06_ = 0.08, *p* = 0.42). Perceived competence was not a significant predictor of intrinsic satisfaction at the between-person level (γ_08_ = 0.07, *p* = 0.27).

Of the control variables, time in study and BMI were not significant predictors of intrinsic satisfaction with exercise class (γ_130_ = 0.01, *p* = 0.30; γ_09_ = -0.01, *p* = 0.87, respectively). Wednesday and Sunday were the only significant day-of-week effects with participants reporting less satisfaction with classes on those days than on Tuesday (γ_150_ = -0.03, *p* < 0.05; γ_190_ = -0.05, *p* < 0.05). Strength (γ_200_ = 0.51, *p* < 0.01) and flexibility (γ_210_ = 0.56, *p* < 0.01) classes also tended to be rated as more satisfying than cardio classes.

Overall, perceptions of the activity, the social climate, and the self of classes explained 63.9% of the variance in participants’ ratings of intrinsic satisfaction with exercise classes. Pseudo R^2^ revealed that of the variance explained, perceived competence accounted for 34% and attraction to group – task accounted for 33% of the explained variance. Instructor encouragement accounted for 24% of the explained variance. Each of the remaining predictors accounted for less than 5% of the variance in intrinsic satisfaction with exercise class.

## Discussion

This study examined dynamic perceptions of the activity, the social climate, and the self to predict fluctuations in exercisers’ intrinsic satisfaction with exercise classes over a 30-week program. Although people were highly satisfied with the classes, people’s satisfaction also tended to fluctuate from class-to-class. This within-person variation has received little attention in previous work, which emphasized between-person differences in intrinsic satisfaction.

Findings were mixed regarding the intensity of exercise classes and intrinsic satisfaction. Interestingly, the negative association, where more intense exercise classes were evaluated as being less intrinsically satisfying, was present at the within-person level but not the between-person level. This finding can be understood as a consequence of the unpleasant feelings which occur during participation in high-intensity exercise as a result of approaching and crossing over the ventilatory threshold (Acevedo et al., 1994; Ekkekakis, 2003). This unpleasant affective response to intense exercise is likely to contrast with hedonic goals of exercise participation and therefore reduces intrinsic satisfaction with the exercise class. Therefore, participants also tended to experience lower intrinsic satisfaction during classes that were perceived as more intense than usual. The exception to this finding was that people were more satisfied with cardio classes that were more intense than usual (as indicated by deviations in both perceived exertion and maximal heart rate). It is possible that exercisers’ goals and expectations regarding the intensity reached during cardio classes were greater compared to other types of classes. Therefore, upon completion of a particularly intense exercise cardio class, goals regarding intensity and challenge were achieved, thus enhancing intrinsic satisfaction with the class. Given that many individuals engage in cardio exercise classes as a way to be active, the time-varying, within-person association found in this study suggest that cardio classes should continually challenge participants. Physically challenging participants more so than usual in a given cardio class will increase intrinsic satisfaction and likely increase adherence. Future research assessing personal goals as well as intensity and exertion of the different types of group exercise class would help untangle associations between perceptions of the class and intrinsic satisfaction.

Intrinsic satisfaction was related to the physiological measure of exercise intensity more so than the self-reported measure of perceived exertion. This suggests that the physiological measure may be more indicative of the physiological response of approaching and exceeding the ventilatory threshold associated with unpleasant feelings than the self-reported measure. These results suggest that self-monitoring exertion may be less valuable than previously believed (e.g., Acevedo et al., 1994), at least when it comes to regulating intrinsic satisfaction.

This is the first study to establish that class-to-class fluctuations in cohesion impact intrinsic satisfaction with exercise classes. Recently, Dunlop et al. (2012) examined changes within elements of cohesion across three exercise class sessions and found that social cohesion elements were dynamic whereas task cohesion elements tended to be static. In contrast, this study revealed that substantial within-person variability in both social and task cohesion elements were present across exercise classes and that class-to-class changes in exercisers’ personal involvement with the group task (i.e., attraction to group-task) were positively associated with intrinsic satisfaction with the exercise class. It may be that as participants progressed through the 30-weeks study, fitness adaptions allowed participants to participate fully and complete all activities within each exercise class. Thus the nature of the task changed with adaptions, participants were able to feel more involved in the task, and as a result found the classes more intrinsically satisfying. Therefore, efforts to enhance intrinsic satisfaction among exercisers are likely to benefit from strategies that continually increase exercisers’ perception of personal involvement with the group task, above typical levels. One way that fitness classes may be able to facilitate these feelings of personal involvement with the task is by offering multiple options for a given exercise so that individuals can adapt their movements to a level that they find appropriately challenging (e.g., presenting exercisers with a push up activity but also presenting a modified version where exercisers can complete the push up activity on their knees). This would allow each individual to participate and engaging physical activity regardless of ability.

Results from our study regarding associations between overall attraction to the group concerning the task as well as social aspects and intrinsic satisfaction align with previous research revealing that the greater the cohesiveness of a group, the greater members’ satisfaction and adherence (Carron et al., 1998; Estabrooks and Carron, 1999). Conversely, previous research among university students enrolled in a group exercise intervention found a negative association between closeness and bonding regarding social aspects of the group and adherence (Spink and Carron, 1994, Study 1). Results from our study provide a potential mechanism for this negative association. It may be that being less integrated in the social aspects of the group allows exercisers to focus on the task at hand, derive satisfaction, and ultimately adherence, from completing the task rather than their closeness to the group.

This study was the first to distinguish between overall differences and class-to-class changes in instructor behavior. Contrary to previous findings linking overall instructor encouragement with feelings of intrinsic satisfaction and enjoyment (Fox et al., 2000; Bray et al., 2005; Edmunds et al., 2008), our findings indicate that overall instructor encouragement has little effect of exerciser’s feelings of intrinsic satisfaction with the class. Instead, exercisers experienced greater intrinsic satisfaction on days when instructors were more encouraging than usual. Therefore, strategies to enhance intrinsic satisfaction via exercise instructors should focus on providing more positive reinforcement and encouragement than is typical. The positive association between overall instructor control and intrinsic satisfaction with the exercise class is surprising given that control can hinder feelings of autonomy and enjoyment (Deci and Ryan, 1985, 2000); however, it may be that individuals who were insufficiently active prior to beginning an exercise program may prefer more technical instruction and feedback from an instructor to properly learn and master skills and techniques used within the exercise class. Offering smaller classes for exercisers that are new to an exercise routine may be particularly useful in providing individuals with personalized and technical instruction and feedback needed to enhance intrinsic satisfaction.

This was also the first study to examine the association between both exercisers’ overall perceived competence and changes in perceived competence and intrinsic satisfaction during classes. Results were contrary to previous findings in youths and young adults. Previous research suggested that exercisers who tend to feel more competent during an exercise class also tend to feel more intrinsically satisfied (Whitehead and Corbin, 1991; Edmunds et al., 2006); however, our results suggest that when simultaneously evaluating both time-invariant and time-varying processes, the time-invariant, between-person association does not predict intrinsic satisfaction. Rather, results from this study reveal exercisers who felt more competent than usual in a given exercise class tended to feel more intrinsically satisfied with the class (a time-varying, within-person process). Perceived competence during a specific exercise class is likely to enhance intrinsic satisfaction by increasing persistence toward and the likelihood of attaining class-specific goals (Elliot and Dweck, 2013). These results suggest exercise classes that emphasize improving upon one’s previous performance more than usual may provide the greatest opportunity to enhance feelings of competence, intrinsic satisfaction, and perhaps ultimately adherence. Exercise classes that offer modified versions of each exercise may provide the best opportunity to enhance feelings of competence because exercisers would have options to choose an exercise that matches or slightly exceeds their ability. Thus when an exerciser successfully completes the activity, they feel competent. Adapting these modifications over time to match advancements would also be important.

### Limitations

First, our sample was fairly homogeneous with respect to age, race, BMI, and previous level of physical activity. Conclusions about the factors which contributed to intrinsic satisfaction with exercise classes in this sample may not generalize to experienced exercisers. However, we believe that the findings from this study provide key insights regarding satisfaction with group-based exercise programs in a demographic group with a great need for such programs (i.e., insufficiently active and overweight/obese adults). The number of participants in the study limited statistical power for detecting between-person associations. This limitation was offset by the intensive within-person assessments, which permitted a powerful analysis of within-person associations between class characteristics and intrinsic satisfaction. Future research that is powered to detect both small between- and within-person associations will extend findings from this study by further untangling associations between perceptions of the activity, the social climate, the self, and intrinsic satisfaction. Participants completed questionnaires immediately following each class; however, some constructs may have changed during classes and required more intensive assessments. We attempted to reduce the impact of this limitation by including an objective measure of exertion. Additionally, we adapted, and sometimes created, measures to assess constructs in the present study. Measures were adapted or created to reduce participant burden that may result from the intensive sampling. The high measurement completion rate (82%) as well as the internal consistency of responses for adapted measures such as intrinsic satisfaction and perceived competence suggest that more burdensome measures may be feasible in future work. Therefore, future work should examine the time-varying nature of these constructs, and their associations with intrinsic satisfaction, using other, more well-established measures [e.g., Intrinsic Motivation Inventory (McAuley et al., 1989)]. Furthermore, because of the possibility of participant burden and fatigue we controlled for time in study in our statistical models to account for any reactivity to study procedures. We recognize that extrinsic goals and extrinsically derived satisfaction can play a pivotal role in exercise classes. While we agree that examining the time-variant and time-invariant influences that regulate both intrinsically and extrinsically derived satisfaction is an important direction for future research, it is beyond the scope of this paper. Furthermore, future research would benefit from assessing daily events that occur outside of the exercise class as such events may color an individual’s evaluations of a class on a given day. Finally, due to the non-experimental design of this study, we are unable to draw conclusions about causality from these data. Experimental work is needed to make strong causal inferences.

## Conclusion

Ultimately, the results from this study indicate several characteristics of exercise classes overall (e.g., cohesion, instructor behavior) and at the class level (e.g., cohesion, instructor behavior, and perceived competence) that can be useful in enhancing intrinsic satisfaction within an exercise class. Designing exercise classes or programs that are intrinsically satisfying to exercisers is important for maintaining behavior (Fleig et al., 2011). These findings lead us to recommend that group exercise classes should be well-organized to provide structure and instructors should consistently promote cohesion. Additionally, from class-to-class, instructors should aim to enhance perceptions of competence (e.g., set appropriate levels of difficulty, provide sincere verbal praise for improvement), and encourage exercisers’ efforts more so than usual. Group-based exercise programs that consider both global influences (i.e., typical class characteristics) as well as micro-influences (i.e., characteristics of individual classes) on exercisers’ satisfaction will increase the likelihood that individuals adopting new exercise programs will be satisfied with individual classes and return to the group exercise environment.

## Conflict of Interest Statement

Dr. Jinger S. Gottschall is the owner of a fitness facility that uses the group exercise program described in this study.
